# Extended incubation recesses in sanderlings are impacted by temperature and body condition

**DOI:** 10.1098/rspb.2023.2264

**Published:** 2024-02-21

**Authors:** Léa Etchart, Nicolas Lecomte, François-Xavier Dechaume-Moncharmont, Jérôme Moreau, Johannes Lang, Thomas Pagnon, Benoit Sittler, Maria Teixeira, Loïc Bollache, Olivier Gilg

**Affiliations:** ^1^ UMR 6249 Chrono-environnement, CNRS, Université de Franche-Comté, 25000 Besançon, France; ^2^ Canada Research Chair in Polar and Boreal Ecology and Centre d'Études Nordiques, Université de Moncton, Moncton, New Brunswick, Canada; ^3^ Univ Lyon, Université Claude Bernard Lyon 1, CNRS, ENTPE, UMR 5023 LEHNA, 69622, Villeurbanne, France; ^4^ UMR 6282 CNRS, Université de Bourgogne, 6 boulevard Gabriel, 21000 Dijon, France; ^5^ Groupe de Recherche en Ecologie Arctique, 21440 Francheville, France; ^6^ Working Group for Wildlife Research at the Clinic for Birds, Reptiles, Amphibians and Fish, Justus Liebig University Giessen, 35392 Giessen, Germany; ^7^ Chair for Nature Conservation and Landscape Ecology, University of Freiburg, Freiburg, Germany

**Keywords:** parental care, incubation strategy, parent–offspring trade-off, sanderling, Greenland

## Abstract

Complex incubation strategies have evolved to solve the trade-off between parent survival and care for their eggs with often brief departures (recesses) that maximize egg survival, and infrequent extended recesses maximizing adult condition. Here we examined incubation behaviour of sanderlings (*Calidris alba*), a species that exhibits both biparental and uniparental incubation behaviour. During 11 breeding seasons in Greenland, we have quantified incubation variability with thermologgers placed in nests. We estimated the impact of environmental conditions and individual characteristics on the occurrence and the duration of recesses. We found that extended recesses are a unique feature of uniparentals, and their frequency and duration increased in colder temperatures. The relationship was mediated by body condition, with individuals in poor condition performing longer extended recesses in colder temperatures. This suggests that extended recesses may represent a shift towards self-maintenance at the expense of the egg care, allowing birds to continue incubating under unfavourable conditions. Our study illustrates how extended recesses may be a key breeding strategy to overcome high energetic costs associated with incubation. Quantifying such behavioural flexibility paves the way for tracking future behavioural responses of individuals in the face of changing environments.

## 1. Introduction

Reproduction requires an important investment of time and energy, and breeding individuals must strike a balance between survival and reproduction during this critical period. In birds, incubation can be one of the most energetically demanding stages of reproduction [1,2], especially in extreme environments [3]. Harsh and unpredictable Arctic environments can for instance induce a high pressure on reproductive individuals. First, the thermoregulation costs for parents are higher than in more temperate regions [4,5]. Second, incubation is more energy-consuming due to faster cooling of the eggs in cold weather, necessitating increased effort from the parent to maintain the optimal temperature at the nest. For income breeders (i.e. unable to rely on energy reserves stored before incubation), a significant conflict arises between incubating the eggs and engaging in essential self-maintenance activities such as foraging [6], drinking [7] or preening [8]. This conflict becomes particularly pronounced for uniparental birds, when only one parent cares for the nest [5].

Unlike species with biparental incubation, uniparental incubators cannot benefit from shared nest duties [9] and must leave their nests more frequently, a behaviour known as a recess [10,11]. Their nests are hence more exposed to predators and cooling [10,12–14]. To maintain a positive energy balance in challenging conditions, birds may employ different strategies. They can either increase the frequency of recess [12] or they can extend the duration of their foraging bouts to compensate for energy loss during inclement weather (see [4,5,15,16]; electronic supplementary material, table S1).

Complex strategies may have evolved within this trade-off between recess frequency and duration. Some species, or some individuals within a species, may favour frequent short recesses, while others may exhibit infrequent prolonged recesses, coined here as ‘extended recesses’. To date, seven studies have focused on understanding and analysing the causes of extended recesses in 11 species with uniparental incubation [4,5,15–19]. While these studies provide first insights into the challenges of detecting and analysing these incubation events, they have also created opportunities for quantifying their duration and elucidating the contributing factors in other species.

Extended recesses refer to instances when incubating individuals depart from their nests for a relatively long period of time (length can vary; see section 2d). These recesses are often considered as rare occurrences or disregarded as measurement artefacts. However, a few studies have already highlighted their occurrence, primarily in relation to prolonged periods of cold or stormy weather [5,16,18,19]. With low temperatures, incubating parents may indeed take longer recesses to fulfil their own energy requirements [20]. However, these extended foraging periods also expose the eggs to fluctuating external temperatures, potentially slowing down or even pausing the embryogenesis [21,22]. As a consequence, delayed hatching and increased exposure to predators can occur [23]. Thus, extended recesses can be viewed as a specific trade-off between adult survival and maintenance on one hand, and egg development on the other, when the typical shorter recesses no longer enable the birds to cope with their environment. In this context, the body condition of the incubating parents may also play a critical role and further modulate this trade-off. Additionally, the differential investment in pre-laying activities between males and females, such as females laying eggs [12], may also influence incubation strategies.

The aim of the study was to assess the occurrence, predictors, and underlying factors of extended recesses on the daily incubation behaviour in an Arctic-nesting shorebird using both bi- and uniparental care during the incubation period, the sanderling (*Calidris alba*). We used 11 years of observational data collected at two high-Arctic study sites. Despite permanent daylight, sanderlings maintain a day-night incubation rhythm. During the ‘night-time’ (i.e. between 17.00 and 09.00), birds incubate almost continuously, taking only few recesses [5] while during daytime (i.e. the warmest period of the day), birds will take advantage of the warm hours to take more recesses. First, we aimed at determining the environmental conditions and individual characteristics leading to the occurrence and duration of extended recesses. In addition, we sought to identify the time interval between the triggering event and the onset of the extended recess, considering the daily trade-off between survival and reproduction. We also conducted a comparative analysis, examining how the environmental conditions and individual characteristics influence the duration of the classical short recesses. Second, we assessed the consequences of extended recesses on the interpretation of the total duration of recesses per day (TDR). TDR is a commonly used proxy (e.g. [6,13]) for studying incubation behaviour, as it directly reflects the nest attentiveness [24]. By analysing TDR, we could compare our findings with previous studies and assess the importance of extended recesses at the daily scale.

As an income breeder, the sanderling heavily relies on its immediate environment for breeding and survival [25]. Given that it breeds in harsh high-Arctic regions and arrives from long-distance migrations with depleted energy reserves, we hypothesized that by breeding at the limit of their physiological capabilities, sanderlings perform extended recesses when under stressful environmental or individual conditions. We predicted: (i) that extended recesses would predominantly occur in uniparental nests, as biparental parents have more time for self-maintenance activities during the incubation period; (ii) that females would exhibit a higher likelihood of performing extended recesses compared to males, considering the energetic demands of eggs-laying; (iii) that the occurrence and duration of extended recesses would increase after periods of cold temperatures due to heightened energetic requirements and reduced activity (i.e. availability) of arthropod prey; and (iv) that an interaction between body condition and climatic conditions should exist, whereby colder periods would have a more pronounced effect on weaker individuals within the population.

## 2. Material and methods

### (a) Study species and sites

The sanderling is a small (44–71 g) long-distance migratory shorebird breeding in the High Arctic (electronic supplementary material, figure S1c) [26]. They usually arrive at their northeast Greenland breeding grounds from late May to mid-June [27]. Upon arrival they switch their diet from small marine invertebrates to terrestrial arthropods (i.e. both insects and spiders). They are generalist insectivores, with a broader diet than other species in this guild (e.g. dunlins, *Calidris alpine*; snow buntings, *Plectophenax nivalis* [28]). This is likely to be beneficial in high-arctic environments, where the abundance of prey is highly variable [29]. For example, some arthropods (e.g. *Tipulidae*, *Chironomidae*) will peak synchronously in early summer, providing short resource peaks for arctic shorebirds, while others (e.g. *Areneae*) are less abundant but available throughout the breeding season [30].

Sanderlings also exhibit a mixed incubation strategy, with both biparental and uniparental care observed, and with both sexes able to incubate and rear chicks [31–33]. We studied sanderlings during 11 consecutive breeding seasons (2011–2021), from mid-June to early August, at two locations in Greenland: Hochstetter Forland (75.15° N 19.70° W) and Karupelv Valley (72.50° N 24° W) (electronic supplementary material, figure S1a). We treated these sites as one population (no difference among them in statistical analyses; see sections 3d–3f).

Winters in northeast Greenland are characterized by very cold temperatures, ranging between −15°C and −25°C. However, during the sanderling's breeding season, temperatures rise above 0°C, with average monthly temperatures between 2°C and 4°C [34]. Both study sites are within the Northeast Greenland National Park, an area with minimal human impact, and are part of the ‘prostrate shrub tundra’ bioclimatic subzone [35].

### (b) Nest monitoring, timing of breeding and ground-level temperatures

Nests were searched in suitable habitats and located opportunistically by flushing incubating adults or by following birds with anti-predator behaviour [36]. For nests discovered during laying, we assumed a laying rate of one egg per day to determine the initiation of incubation (with an average of 4 eggs per nest) [37]. To estimate the first day of incubation for nest with complete clutches, we employed three approaches. This involved using the hatching time recorded with thermologgers (see below), direct observations of hatching eggs or young in the nest cup (with the mean incubation period of the species subtracted), or by floating the eggs [38] (electronic supplementary material, figure S2). We used the incubation starting date to estimate the age of the clutch throughout the entire incubation period.

Ground-level temperature was determined as in [11]. We used the temperatures recorded at one-minute intervals from inactive nests, which included nests that were deserted, predated or hatched, within the same site and similar habitat (see following section for details). These records provided representative measurements of the daily and hourly ground-level temperature specific to the sanderling breeding microhabitat at each site.

### (c) Incubation behaviour and incubation strategies

In each nest, we monitored incubation behaviour using a temperature probe (Flylead Thermistor PB 5009 with 60 cm cable) coupled to a data logger (TinyTag Plus2 TGP-4020; Gemini Data Loggers Inc., West Sussex, UK; electronic supplementary material, figure S1d; see method elsewhere, e.g. [10,11]). Probes were set to record temperature every minute.

Uniparental and biparental incubation strategies were assigned to each nest following [39], and only uniparental nests were used in the following analyses (see section 2e). This approach uses a discriminant equation based on the daily number and duration of recesses observed in nests with known strategies. It has been shown to reliably assign the incubation strategy of sanderlings (i.e. 99% after 24 h and 100% after 4 days of temperature recording).

### (d) Quantifying recesses

Incubation recesses have already been investigated in many bird species, including sandpipers [5,10,11,15]. These recesses are fundamental for birds to pause their incubation, allowing them to forage in order to rebuild their energetic reserves. For most species, these recesses tend to occur during the warmest periods of the day [11], i.e. when arthropods availability is the highest [24]. Being ectotherms, arthropods availability is known to be linked with temperatures, wind speed, or precipitations [40,41]. In some instances, long recesses lasting from one to 72 h have been reported (e.g. [42]). However, a standardized method for detecting and quantifying these extended recesses has yet to be established.

In our study we only considered recesses longer or equal to three minutes to account for the possible uncertainty of 1–2 min around the documentation of the exact onset and ending times of a recess. To score and measure the duration of each recess, we employed a modified method based on previous studies. Instead of considering a recess when the nest's temperature had fallen by 3°C or more from the median incubation temperature over a 24 h period [10,11,39,43], for days with median incubation temperature above 36°C (in order to filter low quality recordings), we defined a recess as a withdrawal characterized by a temperature drop of 4.5°C from the maximum temperature over a 24-hour period. While both methods yield similar results for short recesses (electronic supplementary material, table S2), the first method is in fact inadequate to document extended recesses as the median nest temperature can drop below 36°C during those periods, regardless of the quality of the recording. Our alternative method, based on maximum temperature values, hence allowed us to include extended recesses in the analysis.

To filter out poor-quality records, we retained nests with ≥24 h monitoring and used a threshold of 37.5°C for the maximum daily ground-level temperature (accounting for the 1.5°C difference mentioned earlier). Furthermore, we removed days with erratic patterns, malfunctioning TinyTags (27 nests) and recesses corresponding to the capture of individuals. From this filtered dataset, we extracted each recess, calculated its duration, and then determined the total daily duration of recesses (TDR), which represents the sum of all recess duration recorded over a 24 h period.

**Figure 1.**
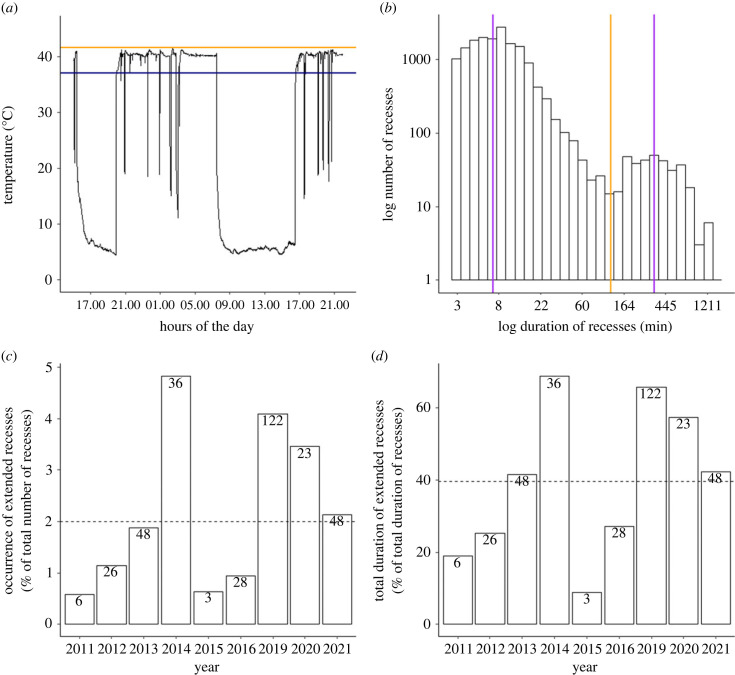
Duration and number of extended recesses recorded on sanderling nests in Greenland (2011–2021). (*a*) Example of a TinyTag recording from Hochstetter site (2019), with two extended recesses of 274 and 544 min between several short recesses of 1–27 min; yellow line: maximum ground-level temperature recorded that day; blue line: temperature threshold (Tmax −4.5°C) used to define recesses. (*b*) Distribution of recess durations (in log-scale); the purple vertical lines represent the two modes of the distribution, 7.1 and 337.5 min). The orange vertical line represents the antimode, 119.3 min discriminating short and extended recesses (see Material and methods). (*c*) Annual proportion of extended recesses (over the total number of recesses). (*d*) Annual proportion of extended recesses' durations (over the total duration of recesses). In both lower panels, the number of extended recesses is given in the bars and dashed lines represent mean proportions over the period 2011–2021 (including 2017 and 2018 with 0 extended recess, but not shown on the panels).

The distribution of recess duration exhibited a distinct bimodal pattern, with extended recesses forming a separate mode on the right side of the distribution (figure 1*b*). To determine an objective threshold (i.e. value above which a recess is considered as ‘extended’) for distinguishing between ‘short’ and ‘extended’ recesses, we used the ‘multimode’ package [44] and retained the antimode as our discriminating threshold (i.e. approximately 120 min, consistent with a previous analysis [4]; figure 1*b*). Furthermore, the bimodal distribution of recess duration precluded its use as a continuous variable in our LMMs (see below), a limitation that also runs counter to the assumption of normal distribution required for our analyses. This statistical categorisation of recesses into shorter and longer duration is inherently tied to our data collection approach, and we posit that this method could be applied in various other sites and species as a means of objectively delineating recess duration. Although the demarcation between shorter and longer recesses near the antimode point may appear somewhat arbitrary, it provides a useful framework for grouping recess duration in a standardized manner.

To estimate the 95% confidence intervals (95% CI) for this threshold, we employed 1000 bootstrap iterations for each year and study site. Furthermore, we compared overlapping 95% confidence intervals in the forest plots to assess significant differences in threshold values among years (electronic supplementary material, figure S3) and/or between study sites (HOCH cut-off value = 111.5 min, CI [96.1;123.7], KVPE cut-off value = 113.3 min, CI [93.2;136.7]) [45,46].

### (e) Body condition

When feasible, incubating birds were caught on their nest with a 40 cm-wide clap net set up over the nest. We took the tarsus length, from the tarsal joint to the distal end of the tarso-metatarsus with digital or dial callipers (±0.1 mm) and body mass using spring scales (±1.0 g with 100 g Pesola spring scales, Pesola AG, Baar, Switzerland). We used these measurements to assess the body condition of 71 incubating individuals using the scaled mass index [47]. Birds were only captured once during the incubation period (mostly during the first half of the incubation) therefore not representing the seasonal changes in body condition, but there was no evidence of change in body mass during the season at the population level (electronic supplementary material, figure S4, table S3). Morphometric measurements were also used to determine the sex of individuals for which molecular sexing was not available (see electronic supplementary material).

### (f) Statistical analyses

Although extended recesses were present in a few biparental nests, their occurrence in these nests was anecdotal (see section 3a). We therefore restricted our analyses to uniparental nests (including the final uniparental period of ‘swap nests’, i.e. biparental nests with desertion of one breeder during the monitoring period) to document the presence, causes and consequences of this behaviour.

We collected the following individual variables: body condition, sex, nest types (uni/swap), and ground-level temperature data. Cold ground-level temperatures may compel birds to remain in the nest until their energy reserves are depleted, leading to extended recesses. Therefore, the pre-recess temperature values could potentially explain the nature (short or extended) and duration of the recess. However, we lacked prior knowledge regarding the most relevant duration of the time window preceding the recess to record ground-level temperature for predicting the nature or duration of recesses. To determine the appropriate response time to ground-level temperature, we conducted two optimization models using different lengths of time windows (i.e. average ground-level temperature measured during 1, 2, 6, 12 and 24 h periods before the onset of the recess; electronic supplementary material, figure S5, table S4). We used the Akaike's information criterion (AIC) to compare the competing models and identified the set of time windows that equally captured our data [48].

We collected ground-level temperature data and individual variables (body condition, sex, uni/swap) for 63 uniparental nests, totalling 8122 recesses. First, we analysed how these variables could predict whether a recess was short (coded as 0) or extended (coded as 1). We used a generalized linear mixed-effect model (GLMM) with a binomial error distribution, a logit link function, and the probability of a recess being either short or extended as the response variable. The fixed variables included body condition, mean ground-level temperature before recess, and sex of the incubating parent. We tested for an interaction between the ground-level temperature and body condition and selected the best models based on *R*^2^ values (electronic supplementary material, table S6). We hypothesized that there would be a cumulative effect, where cold conditions negatively impact all individuals but have a greater one on those in poor body condition. In addition, we included three covariates: incubation date (i.e. number of days since the beginning of incubation) to control for the incubation stage; periods of the day (day/night) to control for a potential circadian rhythm, and type of nest (uni/swap) to control for nest strategies. Nest identity was included as a random variable to account for the repeated measure design.

Second, we assessed the effect of environmental and intrinsic variables on the duration of (a) extended (*n* = 167 recesses for 37 nests) and (b) short recesses (8345 recesses for 64 nests). The response variable (duration of the recess) was integrated in a linear mixed-effect model (LMM) with an identity link function (Gaussian family). The fixed variables included body condition, mean ground-level temperature before recess, and sex of the incubating parent. We tested for an interaction between ground-level temperature and body condition and selected the best model based on *R*^2^ values (electronic supplementary material, table S6). We also incorporated incubation date and nest type as covariates. Nest identity was included as a random variable to account for the repeated measure design.

In a second set of analyses, we investigated the consequences of extended recesses on the total duration of recesses per day (TDR). Here we discriminated between days with (135 days on 35 nests) or without (156 days on 50 nests) extended recesses. Using LMM with an identity link (Gaussian family), we examined the effects of mean daily ground-level temperature, sex, body condition as fixed factors, along with incubation day and uni/swap as covariates. For both models, we tested for an interaction between ground-level temperature and body condition and selected the best model based on their *R*^2^ values. Nest identity was included as a random variable to account for the repeated measure design.

In all models with an interaction term (electronic supplementary material, table S6), we estimated the value of body condition upon which the relationship between the response variable and the temperature was no longer significant with the ‘simple slopes analysis' of the ‘jtools’ package and Johnson–Neyman intervals [49]. We used three body condition values (minimal, maximal and population median) to calculate the conditional slope of the temperature predictor. To properly manage Type I and II error rates, we applied the false discovery rate adjustment as suggested in [50].

We conducted statistical analyses using R freeware version 4.1.1 [51]. We used the ‘lme4’ package [52] for both GLMM and LMMs. *p*-values for LMMs were obtained using t-tests with the Satterthwaite's method for calculating degrees of freedom (package lmerTest [53]). We used the ‘jtools’ package [49] to perform the ‘simple slopes analysis', with the sim_slopes function. Numerical explanatory variables were scaled (Z-scored) and assumptions of normality and homogeneity of variance were assessed through visual inspection of the residuals.

## 3. Results

### (a) Recess detection, duration and numbers

During the 11 years of the study, we discovered 286 nests and continuously monitored 251 of them using TinyTags. A total of 16 518 recesses were documented from the 170 nests that provided usable data, resulting in a dataset covering 1380 nest-days (electronic supplementary material, table S5). Incubation recesses were ubiquitous as every nest had at least one recorded recess, with 4160 recesses documented in biparental nests (*n* = 67) and 16 518 in uniparental and swap nests (*n* = 103). On average, uniparental and biparental sanderlings took 23.6 and 9.6 recesses per 24 h period respectively (ranges 1–68 and 1–32).

We only detected 14 extended recesses (i.e. 0.2% of the total number of recesses) in 10 biparental nests, compared to 340 in uniparental and swap nests (i.e. 2% of the total number of recesses [=16 518]; see dotted line in figure 1*c*). Among the 103 uniparental and swap nests, 60 recordings (58%) included at least one extended recess during incubation, whereas only 10 out of 67 biparental nests (15%) did. Extended recesses were observed in all years, except for 2017 when monitoring duration was the shortest, and in 2018, when only few nests were found due to very late snowmelt (electronic supplementary material, table S5).

Extended recesses lasted from 120 (i.e. our fixed minimum; see section 2d) to 1353 min (electronic supplementary material, figure S6; mean: 391; median: 340). Despite being rare (figure 1*c*), extended recesses greatly contributed to the total duration of recesses documented in most years (figure 1*d*).

### (b) Optimization models

Our two optimization models returned different time steps. For the probability of extended recesses occurrence, the best model clearly selected ground-level temperatures averaged over the 12 h preceding the recess (electronic supplementary material, figure S5a, table S4a; marginal *R*^2^ = 0.17, conditional *R*^2^ = 0.44, i.e. coefficient of determination for generalized linear mixed models [54]). For the duration of extended recesses, the models with ground-level temperatures averaged over the six hours (electronic supplementary material, figure S5b, table S4b; marginal *R*^2^ = 0.10, conditional $R_{c}^{2}=0.30$) and over the two hours (marginal *R*^2^ = 0.098, conditional $R_{c}^{2}=0.31$) preceding the recess were equally supported (ΔAIC = 2 [48]). The one-hour model was only slightly different from the two-hour model (electronic supplementary material, table S4B). For clarity, we presented the results for six-hour time step, but all steps are discussed below (see section 3d).

### (c) Probability of occurrence of an extended recess

**Figure 2.**
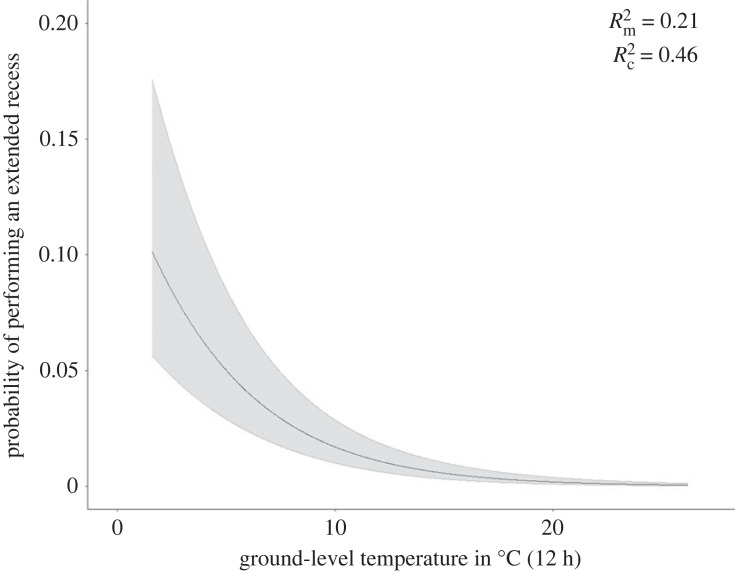
Probability of sanderlings performing an extended recess over a short recess according to the ground-level temperature averaged for the 12 h preceding the beginning the recess in Greenland (2011–2021). The shaded area shows the 95% confidence interval. $R_{m}^{2}$ and $R_{c}^{2}$ correspond to the marginal and conditional *R*^2^ respectively.

Considering the full range of recorded ground-level temperatures (1.6–26.2°C), our top-ranked GLMM model predicted that the probability of taking an extended recess decreased by an average of 20% for each increase of one degree Celsius (marginal *R*^2^ = 0.21; conditional *R*^2^ = 0.46; 8122 recesses for 63 nests; figure 2). The interaction between temperature and body condition was not included in our top-ranked model (electronic supplementary material, table S6). The other model variables (body condition, sex, nest age, uni/swap, period of the day) did not impact the occurrence of extended recesses (electronic supplementary material, table S7).

### (d) Duration of extended recesses

**Figure 3.**
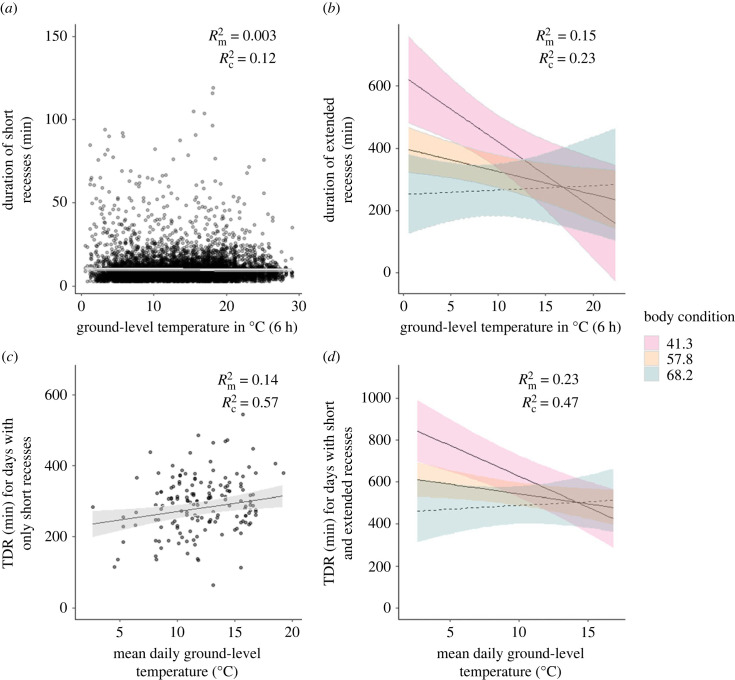
Changes in the duration of sanderling recesses for the range of ground-level temperatures measured in Greenland (2011–2021). Upper panels present the effect of the ground-level temperature averaged over the 6-hours preceding the beginning of a recess on the duration of (*a*) short recesses and (*b*) extended recesses in sanderling. Lower panels present the effect of the mean daily ground-level temperature on the total duration of recesses per day (TDR) for: (*c*) days with only short recesses and (*d*) days with a mix of short and extended recesses. The model fit is in solid line, 95% CI in shading, and dots are raw data. Panels (*b*) and (*d*) are the results of an interaction between the respective ground-level temperature and the body condition. Three different body conditions (min, median, max) are represented to describe the range of body conditions existing in the population. Dashed lines in panels (*b*) and (*d*) present nonsignificant interactions between ground-level temperature and body condition (for individuals with highest body condition). Annotated $R_{m}^{2}$ and $R_{c}^{2}$ correspond to marginal *R*^2^ and conditional *R*^2^, respectively.

Our top-ranked linear mixed model included the interaction between body condition and ground-level temperature averaged over the 6-hours preceding the recess (electronic supplementary material, table S6). This model predicted that the duration of an extended recess decreased as ground-level temperature and body condition increased ($R_{m}^{2}=0.15$; $R_{c}^{2}=0.23$; 167 extended recesses in 37 nests; figure 3*b*; electronic supplementary material, table S8). On average, this model also detected a possible interaction between the two predictor variables; and figure 3*b* presents the model predictions for the minimum (41.3), median (57.8) and maximum (68.2) values of body condition, illustrating the impacts of ground-level temperature on the duration of extended recesses across the range of possible body conditions.

For individuals with the lowest body condition, the model predicted an average decrease of approximately 21 min in the duration of an extended recess for each one-degree Celsius increase in ground-level temperature, spanning a range of 0.6–22.4°C. The decrease was three times smaller (around 7 min per degree Celsius) for individuals with median body condition, while individuals with the highest body condition showed a lengthening of approximately 1 min per degree Celsius.

The ‘simple slopes analysis’ and the Johnson–Neyman intervals, which accounts for the false discovery rate adjustment, revealed that the relationship between duration of extended recesses and ground-level temperature was no longer significant above a body condition of 58.5. This indicates that ground-level temperature impacts the duration of extended recesses for more than half of the breeding adults in this population (i.e. body condition less than 57.8). The other model variables (sex, nest age, uni/swap) included in the model did not influence the duration of extended recesses (electronic supplementary material, table S8).

### (e) Duration of short recesses

To assess whether recess duration was related to preceding ground-level temperatures, we first ran models using ground-level temperature averaged at three different time scales: 1, 2 and 6 h preceding the beginning of the recess. However, the results showed no significant differences. Consequently, we focused on the six-hour model, aligning with the extended recesses' model mentioned earlier. Our top-ranked linear mixed model excluded the interaction between ground-level temperature and body condition (electronic supplementary material, table S6). This model predicted only small variations in the duration of short recesses ($R_{m}^{2}=0.003$; $R_{c}^{2}=0.12$; 8354 recesses for 64 nests; figure 3*a*; electronic supplementary material, table S9). Only nest age influenced the duration of recesses. For each incubation day, recess duration lengthened by ca 5 s. The other model variables (temperature, body condition, sex, uni/swap) had no impact on short recesses duration (electronic supplementary material, table S9).

### (f) Total duration of recesses per day

On average, the TDR was approximately 596 min for the days with extended recesses, but half as long for days without (approximately 288 min; figure 3*c,d*). The top-ranked TDR LMM model for days with both short and extended recesses (135 days, 35 nests) included the interaction between ground-level temperature and body condition ($R_{m}^{2}=0.23$; $R_{c}^{2}=0.47$; electronic supplementary material, table S6; table S10; figure 3*d*). This model predicted a decrease in TDR with the mean daily ground-level temperature, especially for birds with poor body condition.

Throughout the full range of recorded ground-level temperatures (2.6–16.87°C), the TDR for the lowest recorded body condition (41.3) decreased by an average of approximately 29 min for every one-degree Celsius increase of temperature (figure 3*d*). By contrast, for birds with a median body condition (57.8), the TDR decreased by only 9 min per degree Celsius, while it increased by approximately 3 min for the highest body condition (68.2), although it was not statistically significant.

The ‘simple slopes analysis’ and the Johnson–Neyman intervals, which accounts for the false discovery rate, indicated that the relationship between the TDR and ground-level temperature was no longer significant for birds with a body condition above 57.6 (i.e. close to the median value of the population). Without adjusting for the false discovery rate, the body condition threshold was 58.1. Hence, for nearly half of the population performing extended recesses, ground-level temperature impacted the TDR. Additionally, the TDR increased by approximately 11 min on average for each incubation day. The other model variables (sex, uni/swap) did not have a significant impact on the TDR (electronic supplementary material, table S10).

Our top-ranked TDR LMM model for days without extended recesses (156 days, 50 nests) did not include the interaction between ground-level temperature and the body condition (electronic supplementary material, table S6). For each increase of one degree Celsius throughout the full range of recorded ground-level temperatures (2.6–19.2°C; figure 3*c*), this model predicted that the TDR lengthened by approximately 4 min on average for days without extended recesses and for each incubation day (Model $R_{m}^{2}=0.14$; $R_{c}^{2}=0.57$; 156 days for 50 nests; electronic supplementary material, table S11). The other model variables (body condition, sex, uni/swap) did not have a significant impact on the TDR (electronic supplementary material, table S8).

## 4. Discussion

Our 11-year study highlights the variability occurring in recess type and duration influenced by environmental and body conditions. Extended recesses are a regular feature in the incubation behaviour of uniparental sanderlings, supporting our first prediction. While our study shows 58% of uniparental nests exhibiting extended recesses, the few other published estimates varied widely, from 7% to 100% (electronic supplementary material, table S1).

Our study also revealed that the likelihood of performing an extended recess decreased by 10% across the entire range of ground-level temperatures (1–26°C), irrespective of body condition. Two other species of shorebirds (i.e. white-rumped sandpiper; *Calidris fuscicollis*; red phalarope; *Phalaropus fulicarius*) showed a similar change of up to 14% for a range of windchill temperatures of −15 to 8.5°C [15]. In spite of variable responses to temperature, our species illustrate the potential for heightened reproductive costs. This assertion gains support from our findings of a combined impact of body condition and temperature on extended recess duration, supporting our predictions. We quantified these individual responses, revealing that individuals with poorer body condition exhibited longer recesses, especially at lower temperatures. Conversely, short recesses duration remained unaffected by both body condition and temperature across a temperature range of 1 to 26°C (figure 3*a*).

Beyond our fine temporal analyses, we employed the TDR proxy to assess incubation behaviour, allowing comparison with previous studies e.g. [5,10,11]. First, sanderlings do not replace short recesses by one long recess per day. The TDR for days with extended recesses was nearly twice as long as the TDR for days without, indicating that extended recesses affect daily nest attendance. Second, our daily-scale findings echoed those at the recesses scale (i.e. warmer temperatures influenced the TDR negatively for days featuring extended recesses). While quantifying TDR is common, limited studies have accounted for extended recesses and recess duration (e.g. [6]). In line with our findings, previous study reported a negative association between air temperature and TDR (albeit without reporting effect sizes) by considering extended recesses, along with a positive relationship without extended recesses, across four arctic shorebird species. To our knowledge, our study is the first to demonstrate a relationship between TDR and body condition across varying air temperatures. This could help re-evaluate TDR metric in previous studies

### (a) Drivers of extended recesses

Ground-level temperature alone could explain the occurrence of extended recesses, but both ground-level temperature and body condition influenced their duration. Cold spells increase energetic pressure for both the parent and the eggs, as parents lose more energy and eggs cool faster when unattended [4,5]. Birds can withstand harsh conditions for some time before initiating extended recesses. Unlike short recesses, mostly taken during daytime, extended recesses happened at any time, both daytime and night-time. Therefore, the exact time of departure for an extended recess may be related to the current energy balance and energy loss during incubation bouts (i.e. two variables that we could not measure), which are reflected in temperature measurements. It is also possible that a certain degree of mass loss due to cold temperatures triggers an extended recess, regardless of the bird's body condition.

While temperature impacts the energy balance, it also impacts food availability via its influence on arthropod abundance and activity [55]. Therefore, under cold conditions, sanderlings may need more time for energy replenishment, especially to compensate for the low availability of arthropods. However, this effect is likely mediated by the body condition, with individuals in better conditions having more energy to invest in reproduction [56,57] and experience fewer energetic constraints than those in poorer condition. While declining temperatures trigger extended recesses regardless of body condition, the duration of extended recesses only increases for individuals below a given threshold of body condition (see section 3d; figure 3*b*). Extended recesses have been poorly studied especially in shorebirds in the past but are likely linked to body condition. Blue petrels (*Halobaena caerulea*) for example, exhibited extended recesses when a certain mass threshold was reached, but when food availability was low, the birds returned to their nest with a reduced body mass [58]. If extended recesses do not provide sufficient gain, individuals face the dilemma of returning to their nest in even poorer condition, lengthening their foraging bouts, or ultimately abandoning their nest. In this case, extended recesses could be detrimental for the adults that did not meet their energetic needs, but also for the eggs. While no study described a potential impact on the chicks' development, extended recesses likely lengthen the incubation period, mechanistically increasing the risk of predation, but probably also reduce embryo viability [16]. Extended recesses are probably not always sufficient for birds to fully replenish depleted reserves and could explain why some individuals in our study performed successive extended recesses. Finally, the presence and duration of extended recesses were not influenced by sex nor nest type (uniparental versus swap) in our study, suggesting that pre-incubation behaviour costs could be balanced between sexes. The absence of difference between the two types of uniparental nests included in our analyses (i.e. uniparental and swap nests) supports our methodological choice to merge these two types of nests in our analyses.

### (b) Timestep influence on extended recesses

In all the studies quoted above, the occurrence and temperatures related to extended recesses were analysed at a daily time scale. In our study, we also investigated the speed of individual responses to temperatures. Although our results can be compared with previous studies, it is important to bear these scaling differences in mind. We demonstrated that the average ground-level temperature measured in the 12 h prior to the start of an extended recess best predicted its occurrence, while a shorter timestep (6 h, 2 h or 1 h) best predicted its duration. The 12 h timestep suggests that some birds can endure harsh conditions for a relatively long period (i.e. 6–12 h) before initiating long recesses. On the other hand, the duration of extended recesses appears to be a more immediate response, with a 6 h timestep being the best predictor (and being not competitively different with the 2 h and 1 h timesteps; electronic supplementary material, figure S5b, table S4b). While their scales were different (incubation, daily and recess), Diez-Méndez *et al*. [59] already stated the importance of studying incubation behaviour at different time scales, as it provides insights into various aspects of this behaviour.

### (c) Perspectives

Extended recesses could allow parents to pursue the incubation, despite harsh environmental and poor body conditions. While some papers described a lengthening of the incubation because of extended recesses (e.g. [17]), and therefore a longer exposition to predation pressure, our dataset did not allow us to explore this question. It would be worth studying this potential lengthening in more detail, as well as the potential impact of extended recesses on predation pressure. To go further in understanding the fluctuations in energy reserves during incubation bouts, we identify the need for quantifying the foraging patterns and distances of adults during recesses along with their body reserves.

Finally, our study highlights the importance of extended recesses as a significant component of the incubation strategy in sanderlings and potentially in other species as well. Investigating this behaviour in other species with different life-history traits, such as body masses, would facilitate species comparisons and contribute to a better understanding of how this behaviour is linked to the biology and physiology of each species.

## Data Availability

Data are available from the Dryad Digital Repository: https://doi.org/10.5061/dryad.v41ns1s3q [60]. Electronic supplementary material is available online [61].
